# Adjuvant-Free Murine Models of Allergic Sensitization to the Major Soybean Allergen Gly m 4

**DOI:** 10.3390/ijms262311695

**Published:** 2025-12-03

**Authors:** Ivan V. Bogdanov, Ekaterina I. Finkina, Alfia G. Kamaeva, Marina S. Krasilshchikova, Tatiana V. Ovchinnikova

**Affiliations:** 1 Science-Educational Center, M. M. Shemyakin and Yu. A. Ovchinnikov Institute of Bioorganic Chemistry, Moscow 117997, Russia; finkina@mail.ru (E.I.F.); ovch@ibch.ru (T.V.O.); 2 Moscow Center for Advanced Studies, Moscow 123592, Russia; 3 Department of Experimental Biology with Vivarium, M. M. Shemyakin and Yu. A. Ovchinnikov Institute of Bioorganic Chemistry, Moscow 117997, Russia

**Keywords:** soybean Gly m 4, murine model of allergy, sensitization potential, allergen-specific antibodies, cytokine profiles

## Abstract

Gly m 4, a soybean PR-10 allergen, is known to trigger systemic allergic reactions. However, the intrinsic sensitizing potential of the allergen remains unclear. Adjuvant-free murine models of sensitization to Gly m 4 might help to investigate mechanisms of a soy allergy and establish relevant in vivo platforms for developing novel allergen-specific immunotherapy strategies. BALB/c mice were sensitized to Gly m 4 via intraperitoneal (i.p.), subcutaneous (s.c.), or intranasal (i.n.) routes, with or without adjuvant (alum or lipopolysaccharide (LPS)). In order to assess sensitization, we evaluated levels of allergen-specific IgE, IgG1, IgG2a, systemic anaphylaxis, rat basophil (RBL) degranulation, and cytokine/chemokine profiles in mouse sera. I.n. exposure with or without LPS proved to be ineffective and did not elicit sensitization. I.p. and s.c. routes of sensitization with and without alum induced a Th2-skewed response, which was demonstrated by high levels of IgE and IgG1, systemic anaphylaxis, and IgE-mediated degranulation of RBL cells. Adjuvant-free i.p. administration led to a shift in cytokine production, with reduced levels of proinflammatory (IL-1α/IL-6) cytokines and increased levels of Th2-associated (IL-13/GM-CSF) ones. Thus, adjuvant-free murine models validated the intrinsic sensitizing capacity of Gly m 4. Moreover, Gly m 4 demonstrated similar immunogenic profiles to Bet v 1 in alum-based models. It is the first evidence that soybean Gly m 4 can induce in vivo allergic sensitization in mice without adjuvants, particularly via i.p. and s.c. routes. Established adjuvant-free murine models offer a relevant tool for studying soy allergy and developing targeted immunotherapy.

## 1. Introduction

The soy *Glycine max* is one of the most widely cultivated legume crops and a significant source of dietary protein across the globe [1]. Due to its high nutritional value and economic importance, soy has become a staple in many diets. In spite of these benefits, soy is also recognized as a clinically relevant food allergen. It was historically included by the Food and Agriculture Organization (FAO) of the United Nations among the so-called “Big Eight” allergenic foods (milk, eggs, peanuts, tree nuts, fish, crustacean shellfish, wheat, and soybeans)—the list of the eight top food allergens responsible for the majority of food allergic reactions worldwide [2]. Soy remains a significant and commonly reported allergen, and its labeling is mandated in many countries under updated regulations that now include a “Big Nine” list (together with sesame).

Among the eight officially recognized soybean allergens listed in the WHO/IUIS Allergen Nomenclature Sub-committee database, Gly m 4, a member of the pathogenesis-related protein family 10 (PR-10), has attracted growing attention due to its clinical relevance [3]. This protein shares low sequence identity (48%), but high structural similarity with the major birch pollen allergen Bet v 1 and is primarily implicated in allergic reactions in individuals previously sensitized to birch pollen [4]. Moreover, Gly m 4 is considered a marker allergen for severe manifestations of a soy allergy, including systemic reactions, whereas most PR-10 proteins are typically associated with milder symptoms, such as oral allergy syndrome (OAS) [5].

With the exception of some PR-10 allergens, Bet v 1 was believed for a long time to act as the primary sensitizer within the PR-10 family, with other homologous allergens contributing merely to secondary, cross-reactive symptoms [6]. However, this paradigm has recently been challenged. Recent research has shown that other PR-10 allergens, including Aln g 1 from alder pollen, may have intrinsic sensitization capacity, pointing to a broader allergenic potential within this protein family [7]. This study raised some important questions regarding the intrinsic sensitizing ability of Gly m 4 and its potential role not only in manifestation, but also in initiation of a soy allergy.

In our previous study we have demonstrated a low cross-reactivity of Bet v 1- and Gly m 4-specific antibodies of different classes [8]. This observation suggests that the soybean allergen Gly m 4 could potentially act as an independent sensitizer of the immune system. Nevertheless, it still remains unknown whether Gly m 4 alone, in the absence of adjuvants or prior pollen sensitization, is capable of initiating sensitization in vivo. This question is of high relevance not only for understanding the development of a soy allergy in birch-endemic regions but also for guiding the selection of allergen-specific immunotherapy (AIT) agents.

Murine models of food allergy are used not only as a preclinical approach to investigate the efficacy of new therapeutic strategies, but also to predict allergenicity of new proteins and to unveil the mechanisms of development of allergic diseases, among others, in studying the sensitizing capacity of food proteins [9]. In particular, adjuvant-free intraperitoneal sensitization protocols have been established to assess whether a given protein possesses intrinsic allergenic potential [10,11]. In our earlier work, we have developed the first mouse model of sensitization to Gly m 4 using subcutaneous injections of the purified allergen with alum as an adjuvant [8] that replicated an established protocol of sensitization with the birch allergen Bet v 1 [12]. The present study aimed at the development of adjuvant-free murine models of sensitization to Gly m 4 and the assessment of whether Gly m 4 alone is sufficient to induce sensitization, which is a crucial question for understanding its intrinsic allergenic potential. To do this, we evaluated the efficiency of different routes of Gly m 4 allergen exposure and compared the impact of two commonly used immune adjuvants—alum and lipopolysaccharide (LPS) in promoting sensitization to Gly m 4. Development of adjuvant-free murine models is essential for clarifying the underlying mechanisms of a soy allergy and for creating relevant in vivo platforms to evaluate preventive or treatment AIT strategies.

## 2. Results

### 2.1. Mice Were Successfully Sensitized to Soybean Allergen Gly m 4 by Intraperitoneal and Subcutaneous, but Not by Intranasal Route

**Figure 1:**
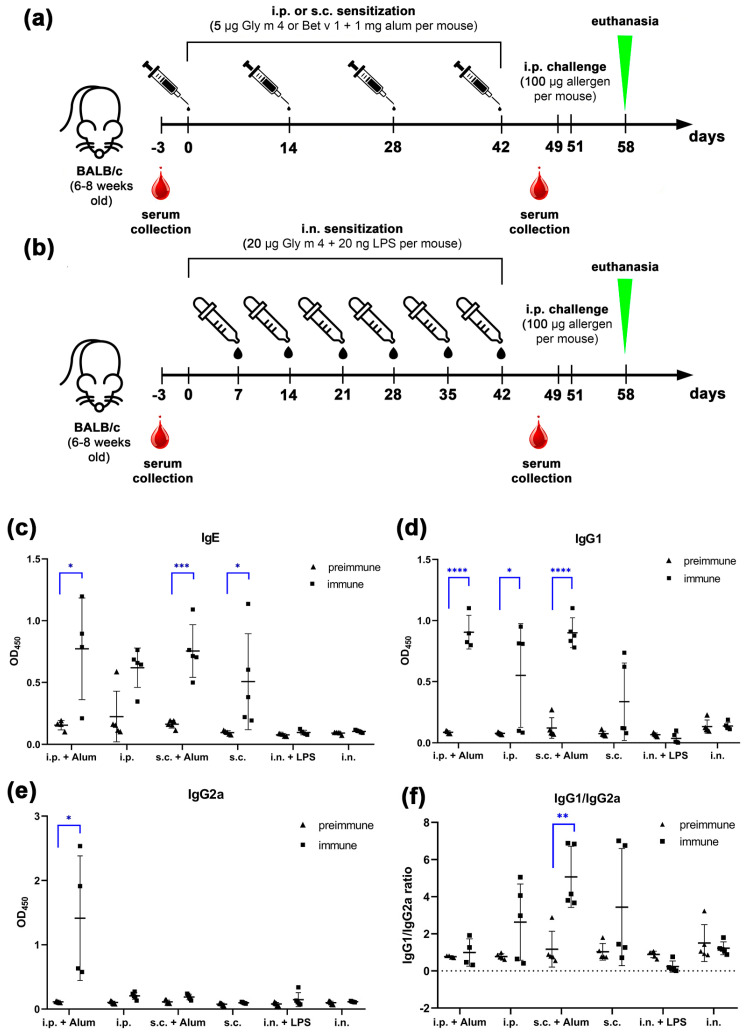
(**a**,**b**) Protocols of mice sensitization with soybean Gly m 4 allergen by intraperitoneal (i.p.) and subcutaneous (s.c.) injections (**a**), or intranasal (i.n.) administration (**b**). (**c**–**e**) Levels of Gly m 4-specific IgE (**c**), IgG1 (**d**), and IgG2a (**e**) in mouse sera before (preimmune) and after (immune) sensitization via different administration routes with or without adjuvant. (**f**) IgG1/IgG2a ratio of Gly m 4-specific antibodies in mouse sera. Significance levels: * *p* < 0.05; ** *p* < 0.01; *** *p* < 0.005; **** *p* < 0.0001.

To investigate the sensitizing capacity of the soybean allergen Gly m 4, we established and compared six murine models of allergic sensitization using wild-type BALB/c mice aged 6–8 weeks. Three routes of allergen administration were tested: intraperitoneal (i.p.), subcutaneous (s.c.), and intranasal (i.n.), each applied both with and without an adjuvant (alum for i.p. and s.c.; lipopolysaccharide [LPS] for i.n.), as shown in Figure 1a,b. Based on previous findings that lower doses of allergen are more likely to induce sensitization [13], whereas higher doses may favor tolerance, mice were sensitized with 5 μg of Gly m 4 per injection via i.p. and s.c. routes. For intranasal administration we reproduced the previously published protocol for the sensitization to the other food allergen, peach Pru p 3, giving intranasally each mouse 20 μg of Gly m 4 per administration [14].

To evaluate the efficiency of sensitization in each model, sera were taken prior to the first sensitization and one week after the final dose. Levels of Gly m 4-specific IgE, IgG1, and IgG2a were measured using ELISA (Figure 1c–e). Our results demonstrated that allergen-specific IgG1 (sIgG1), a marker of Th2-type responses, was significantly elevated (*p* < 0.05) in mice sensitized via the s.c. or i.p. routes in the presence of alum, compared to their preimmune sera (Figure 1d). Serum levels of Gly m 4-specific IgE (sIgE), which are directly involved in the pathogenesis of allergic reactions, were also significantly increased in the same groups (*p* < 0.05; Figure 1c). These results confirm successful induction of a Th2-skewed immune response in both adjuvant-based models.

In the absence of alum, sIgG1 and sIgE levels were also elevated following i.p. and s.c. sensitization, although the antibody titers were generally lower and showed considerable inter-individual variability (Figure 1c,d). The obtained data suggest that Gly m 4 possesses intrinsic immunogenic properties capable of inducing specific antibody responses even without exogenous adjuvant, although sIgG1 and sIgE levels in this case were lower.

In contrast, mice sensitized via the intranasal route, either with or without LPS, failed to induce significant levels of Gly m 4-specific IgG1, IgE, or IgG2a. These results indicate that intranasal administration of Gly m 4 under the tested conditions is unable to induce sensitization and elicit systemic humoral responses.

Interestingly, in addition to Th2-associated antibodies, in the case of i.p. sensitization with alum, we observed a significant (*p* < 0.05) increase in Gly m 4-specific IgG2a (sIgG2a), a subclass associated with Th1-type responses and potential blocking activity (Figure 1e). IgG1/IgG2a ratio of Gly m 4-specific antibodies showed statistical significance only in the case of s.c. sensitization with alum (Figure 1f).

To characterize the sensitizing capacity of Gly m 4, we compared its immunogenicity with that of Bet v 1, the major birch pollen allergen. Using the same sensitization protocol and adjuvant alum, mice were sensitized via s.c. or i.p. administration of Bet v 1. High titers of Bet v 1-specific IgG1 were detected in both groups (Supporting Information, Figure S1), indicating robust Th2-polarization. In the case of Bet v 1-specific IgE, we observed the same high variability between animals within each group as for Gly m 4. In both models, IgG2a responses to Bet v 1 were observed, with higher titers found in the i.p.-sensitized group. The obtained results suggest that soybean Gly m 4, like birch pollen Bet v 1, can induce both Th2-type and partially Th1-type antibody responses when administered with alum.

### 2.2. Gly m 4-Induced Systemic Anaphylaxis and Effector IgE Functionality in Sensitized Mice

In order to confirm the sensitization state of mouse immune system, we evaluated the ability of Gly m 4 to induce systemic anaphylactic reactions in vivo. One week after the last sensitization, all mice were challenged intraperitoneally with adjuvant-free Gly m 4 (100 μg per mouse). After the challenge, the mice were observed for 40 min for symptoms of systemic anaphylaxis, which were rated on the clinical scale provided in Section 4.

**Figure 2:**
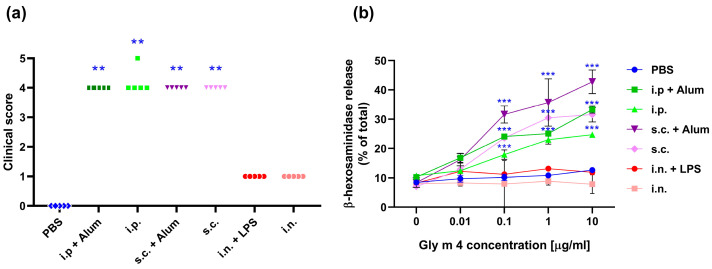
(**a**) Clinical score of systemic anaphylactic reactions after challenge of sensitized mice with Gly m 4. The significance level is indicated in comparison with the control mice: ** *p* < 0.0001. (**b**) Rat basophil (RBL) degranulation assay. RBL cells were preincubated with pooled mouse sera. Degranulation of RBL cells loaded with mouse IgE was induced with Gly m 4 (0–10 μg/mL) and measured by β-hexosaminidase release: *** *p* < 0.000001.

All of the animals sensitized to Gly m 4 by intraperitoneal or subcutaneous routes with and without an adjuvant showed clear signs of systemic anaphylaxis (Figure 2a). These included pronounced reductions in mobility, with all animals displaying no reaction even after prodding, which indicates severe systemic responses. Some animals experienced cyanosis around the mouth. On the other hand, mice sensitized with Gly m 4 with and without LPS via intranasal administration did not show any signs of severe anaphylaxis. This further proves that this route did not cause sensitization under the conditions used in this study.

Statistical analysis revealed a significant difference in anaphylaxis scores between the sensitized groups (i.p. and s.c.) and the PBS-treated control group (*p* < 0.05), confirming the clinical relevance of the observed reactions. Similar anaphylactic reactions have been observed in the case of mice sensitized with Bet v 1 in the presence of alum and subsequently challenged with birch Bet v 1.

The severity of the anaphylactic reactions in mice sensitized with adjuvant-free Gly m 4 was quite similar to that observed in mice sensitized with the allergen administered with alum (Figure 2a). These data suggest that Gly m 4 has intrinsic allergenic potential and is able to induce the sensitization of the immune system and elicit robust systemic reactions without the need for an exogenous adjuvant.

To assess the functional relevance of Gly m 4-specific IgE, raised during the sensitization step, we carried out a commonly used in vitro degranulation assay that uses rat basophilic leukemia cell line RBL-2H3, which expresses FcεRI, a high-affinity receptor for IgE, and is able to degranulate upon cross-linking by allergen-IgE complexes [15]. RBL-2H3 cells were passively sensitized with sera pooled from mice within each experimental group and then stimulated with serial 10-fold dilutions of Gly m 4 ranging from 0.01 to 10 μg/mL.

In support of in vivo challenge results, RBL-2H3 cells, passively sensitized with sera from mice received Gly m 4 via i.p. or s.c. injections, exhibited significant degranulation in response to Gly m 4 (Figure 2b), which confirmed the presence of functionally active allergen-specific IgE. In contrast, sera from mice sensitized via intranasal administration failed to induce detectable degranulation, confirming the conclusion that this route did not induce sensitization in our study.

Interestingly, a slightly higher degree of degranulation was demonstrated for RBL cells sensitized with sera from the s.c. group compared to the i.p. group. This finding is in line with previous reports for Bet v 1, where specific IgE raised upon subcutaneous administration demonstrated enhanced functional activity in RBL-2H3 assays [16]. These results suggest that, while both i.p. and s.c. routes are capable of inducing systemic IgE-mediated sensitization, the subcutaneous route may generate IgE with higher biological activity in terms of effector cell activation; however, this claim requires further verification.

Taken together, our results indicate that sensitization to Gly m 4, particularly via subcutaneous and intraperitoneal routes, results in a production of functional specific IgE that can promote both the activation of effector cells and systemic anaphylaxis.

### 2.3. Immune Response Profiling upon Adjuvant-Free and Alum-Based Sensitization to Gly m 4

In order to characterize the immune responses in the established murine models of sensitization to the soybean allergen Gly m 4, we determined cytokines, chemokines, and growth factors in the sera of sensitized mice using multiplex technology. This approach allowed us to assess at a protein level 32 different analytes involved in various aspects of immune regulation, including pro- and anti-inflammatory signaling, leukocyte recruitment, and tissue remodeling.

**Figure 3:**
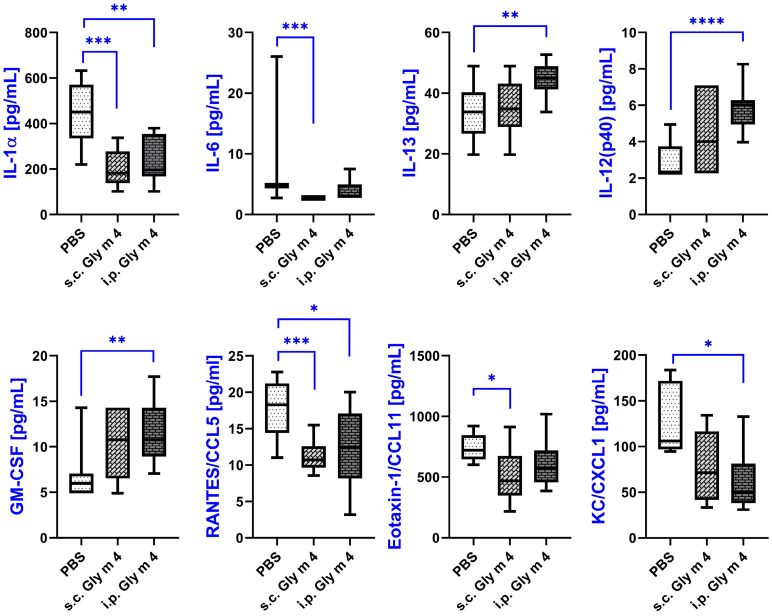
Cytokine levels in sera of mice sensitized by adjuvant-free soybean Gly m 4, measured by multiplex technology. Cytokine levels in mice from the same group are shown as box-and-whisker plots. Significance levels: * *p* < 0.05; ** *p* < 0.01; *** *p* < 0.005; **** *p* < 0.001.

First, we decided to characterize adjuvant-free mouse models of sensitization with soybean allergen Gly m 4. Among the analytes assessed, only eight cytokines and chemokines showed significant differences in mice sensitized with Gly m 4 without adjuvant (either s.c. or i.p.) when compared to the PBS-treated control group (Figure 3; Supporting Information, Table S1).

A marked decrease in the levels of interleukin-1 alpha (IL-1α), a key proinflammatory cytokine, was observed in both sensitized groups. In the PBS control group, IL-1α levels reached an average of 450 pg/mL, whereas in the s.c. Gly m 4 group they were significantly reduced to 181 pg/mL (*p* = 0.0003), and in the i.p. group to 196 pg/mL (*p* = 0.0026), which may suggest a suppression of acute inflammatory responses during sensitization.

A decrease was also observed in the case of pleiotropic cytokine IL-6, which plays roles in both pro- and anti-inflammatory pathways. Although IL-6 concentrations were reduced in both Gly m 4 groups compared to control group, this decrease reached statistical significance only in the s.c. group (*p* = 0.0002), possibly reflecting route-dependent differences in immune modulation.

The anti-inflammatory cytokine IL-13, often associated with Th2-type allergic responses and airway hyperresponsiveness, demonstrated a mild, non-significant increase in the s.c. group (*p* = 0.73), while in the i.p. group, a significant elevation was detected, with mean levels rising from 33.82 to 45.02 pg/mL (*p* = 0.0033). This suggests a systemic Th2 shift, more pronounced upon intraperitoneal sensitization.

IL-12 (p40), a subunit shared by both IL-12 and IL-23, showed a statistically significant increase in the i.p. Gly m 4 group (*p* < 0.0001), although the absolute serum concentrations remained relatively low across all groups. Given its role in driving Th1 differentiation, this increase may reflect a complex interplay between opposing Th1/Th2 pathways during sensitization.

Granulocyte-macrophage colony-stimulating factor (GM-CSF), which is involved in eosinophil activation and allergic inflammation, was elevated in both sensitized groups. However, the increase reached significance only in the i.p. Gly m 4 group, with GM-CSF levels rising from 5.98 to 10.77 pg/mL (*p* = 0.0023).

Among the chemokines, RANTES/CCL5, known for its chemoattractant activity toward monocytes, T helper cells, and eosinophils, was significantly downregulated in both sensitized groups. Its levels decreased from 18.31 pg/mL in the control group to 10.72 pg/mL (*p* = 0.0002) in the s.c. group and to 12.46 pg/mL (*p* = 0.0177) in the i.p. group, which may indicate altered leukocyte recruitment during the sensitization process.

Decreased production was also observed in the case of eotaxin-1/CCL11, a chemokine selectively attracting eosinophils and playing a central role in allergic responses. In the s.c. Gly m 4 group, CCL11 levels declined significantly from 720 to 467 pg/mL (*p* = 0.042), whereas in the i.p. group, the reduction to 569 pg/mL did not reach statistical significance (*p* = 0.072).

Lastly, CXCL1/KC, a potent neutrophil chemoattractant, was diminished in both groups. Although the downward trend was observed for both s.c. and i.p. Gly m 4 sensitization routes, a statistically significant reduction was only detected in the i.p. group (from 106.2 to 49.7 pg/mL, *p* = 0.011).

Taken together, our data indicate that sensitization with Gly m 4 in the absence of adjuvant induces changes in cytokine and chemokine secretion, involving both pro- and anti-inflammatory mediators, and that the route of allergen administration probably influences the strength of the immune response. Notably, these changes reflected both downregulation of major inflammatory mediators and upregulation of specific cytokines commonly associated with allergic inflammation.

To further characterize the immune responses induced by sensitization to the soybean allergen Gly m 4, we analyzed serum cytokine profiles in mice sensitized by s.c. or i.p. injections of the allergen in combination with alum as an adjuvant. Sera of mice sensitized via i.p. and s.c. routes with the birch pollen allergen Bet v 1 plus alum were used for the comparison of the immune responses. Mice that received intranasal administration of Gly m 4 were excluded from the analysis, as this route did not lead to successful sensitization.

**Figure 4:**
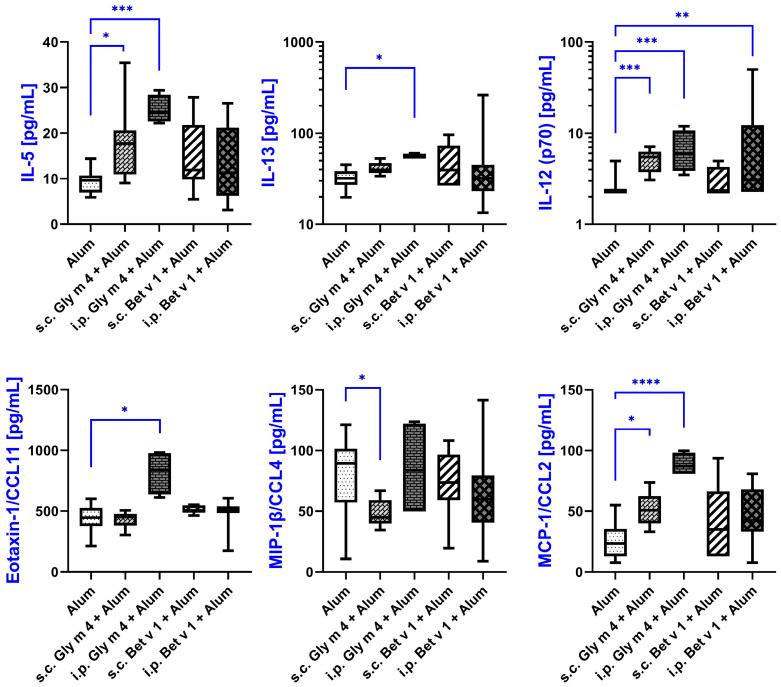
Cytokine levels in sera of mice sensitized by soybean Gly m 4 and birch Bet v 1 with alum adjuvant, measured by multiplex technology. Cytokine levels in mice from the same group are shown as box-and-whisker plots. Statistical analysis was performed using Kruskal–Wallis test with False Discovery Rate (FDR) correction according to the Benjamini-Krieger-Yekutieli procedure. Significance levels: * *p* < 0.05; ** *p* < 0.01; *** *p* < 0.005; **** *p* < 0.001.

Out of the 32 analytes assessed by multiplex analysis, only 6 showed statistically significant differences in at least one sensitized group compared to the control group that received alum alone. These changes point to a selective immune response associated with sensitization to Gly m 4 and, to a lesser extent, to Bet v 1. Higher variability between absolute levels of most cytokines was demonstrated in groups of mice sensitized with Bet v 1 (Figure 4; Supporting Information, Table S1).

Interleukin-5 (IL-5) and interleukin-13 (IL-13), two crucial cytokines of the Th2 immune response involved in the development and pathogenesis of allergic diseases, were elevated in both s.c. and i.p. Gly m 4-sensitized groups. IL-13 levels increased from 32.01 pg/mL in the alum-treated control group to 39.39 pg/mL in the s.c. group (*p* = 0.10) and to 54.65 pg/mL in the i.p. group (*p* = 0.0074), suggesting a more robust Th2-skewing via the i.p. route. IL-5 levels also rose significantly, from 9.6 pg/mL in control group to 11.93 pg/mL in the s.c. group (*p* = 0.0116) and to 11.33 pg/mL in the i.p. group (*p* = 0.001), which confirmed activation of Th2-related pathways during Gly m 4 sensitization.

In addition to Th2-cytokines, we have observed a significant increase in production of IL-12 (p70), which is typically associated with Th1-type responses and immune regulation. IL-12 (p70) levels increased from 2.26 pg/mL in control group to 5.47 pg/mL in the s.c. Gly m 4 group (*p* = 0.001) and to 6.02 pg/mL in the i.p. Gly m 4 group (*p* = 0.0043), indicating a possible mixed immune profile. Interestingly, mice sensitized to Bet v 1 via the i.p. route also showed a modest but significant increase in IL-12 (p70) levels (3.06 pg/mL, *p* = 0.0193), highlighting some degree of overlap in the immune responses induced by the two allergens.

Among chemokines, Eotaxin-1 (CCL11), a potent chemoattractant for eosinophils, was significantly elevated only in the i.p. Gly m 4 group. Its concentration rose from 447 pg/mL in alum-treated control group to 837 pg/mL in the i.p. sensitized group (*p* = 0.0006), which may suggest an enhanced eosinophilic recruitment upon intraperitoneal exposure to Gly m 4.

MCP-1/CCL2, monocyte chemoattractant protein-1, was also upregulated in response to Gly m 4 sensitization. Levels of CCL2 were shown to be increased from 23.46 pg/mL in the control group to 50.82 pg/mL in the s.c. group (*p* = 0.012) and to 87.18 pg/mL in the i.p. group (*p* = 0.0002), indicating a recruitment of monocytes, which may potentially contribute to local tissue inflammation and antigen presentation.

In contrast, macrophage inflammatory protein-1β (MIP-1β/CCL4), a chemokine involved in the recruitment of T cells and macrophages, was significantly decreased in the s.c. Gly m 4 group. Its serum concentration dropped from 89.61 pg/mL in control mice to 44.97 pg/mL (*p* = 0.0116), suggesting a downregulation of specific leukocyte trafficking pathways. In the i.p. Gly m 4 group and Bet v 1-sensitized groups CCL4 levels were more heterogeneous with no significant changes compared to control group.

In summary, our findings demonstrate that sensitization to Gly m 4 with adjuvant alum induces statistically significant cytokine and chemokine changes in mice. Based on the cytokine profiles, the intraperitoneal route seems to boost a more pronounced allergic Th2-type response, potentially making this model more suitable for modeling systemic allergic sensitization in vivo.

## 3. Discussion

The soybean allergen Gly m 4, a member of the pathogenesis-related protein family 10 (PR-10), is the major soy allergen, associated with severe allergic reactions to soy, particularly in patients sensitized to the birch pollen allergen Bet v 1 [5]. Despite high clinical relevance of Gly m 4, data on its intrinsic sensitizing potential is limited. In order to study sensitization potential in vivo, we used inbred mice strain BALB/c. Murine models are widely used to evaluate the sensitization potential of food allergens due to their immunological similarity to humans, short reproductive cycle, and well-characterized immune responses [17]. For instance, adjuvant-free sensitization protocols with intraperitoneal administration have been established to assess an intrinsic allergenic potential of a given protein [10,11].

In our previous study, we developed the first murine alum-based model of sensitization to Gly m 4 to investigate antibody cross-reactivity between two PR-10 allergens—soybean Gly m 4 and birch Bet v 1 [8]. The sensitization protocol included subcutaneous injections of the purified allergen with alum and it replicated an established model of sensitization with birch Bet v 1 [12]. However, the model lacks the ability to reveal the intrinsic allergenicity potential of soybean allergen Gly m 4, as alum can promote the sensitization to non-allergenic proteins due to Th2 responses. This is why, in the current study, we aimed at the assessment of sensitization capacity of the soybean allergen Gly m 4 with or without one of two commonly used immune adjuvants, using different routes of Gly m 4 administration (intraperitoneal, subcutaneous, or intranasal). The developed mouse models of sensitization to Gly m 4 were characterized by measuring levels of Gly m 4-specific IgE, IgG1, and IgG2a upon sensitization, assessing the functional capacity of sIgE to induce degranulation of basophilic RBL-2H3 cells, and by determination of cytokine levels in sera of sensitized mice.

Our results indicate that i.p. and s.c. administration of Gly m 4, both with or without alum, successfully induced sensitization in BALB/c mice. Th2-type responses in these models were demonstrated by elevated levels of allergen-specific IgE and IgG1 [18]. Interestingly, the adjuvant-free models also induced significant Th2-type responses, however, with slightly lower titers and higher heterogeneity compared to alum-based models (Figure 1c,d). This suggests that Gly m 4 has an intrinsic capacity to initiate sensitization, particularly when administered via i.p. and s.c. sensitization ways. The ability of Gly m 4 to induce systemic anaphylaxis and IgE-mediated degranulation of RBL-2H3 cells also supports its clinical relevance and intrinsic allergenic potential.

In contrast, the intranasal administration route, both with and without LPS, failed to induce sensitization. For the i.n. sensitization we used a previously developed sensitization protocol, which allowed us to successfully induce sensitization to the food peach allergen Pru p 3, a lipid transfer protein, when administered together with LPS [14]. By using the same sensitization protocol, we showed that the soybean allergen Gly m 4 failed to induce sensitization via this route, which might reflect structural or biochemical differences between PR-10 and lipid transfer protein allergens. The obtained results indirectly indicate that the i.n. route may be less effective for certain allergens due to particular factors, e.g., mucosal barriers.

By comparing mouse models of sensitization to the major birch pollen allergen Bet v 1, a well-characterized PR-10 protein, we have shown some similarities in the immune responses elicited by both allergens when administered with alum. Both soybean Gly m 4 and birch Bet v 1 allergens led to production in vivo comparable levels of allergen-specific IgE, IgG1, and IgG2a, which suggested that these allergens have similar immunogenic properties in terms of Th2-polarization and systemic reactivity. However, the presence of significant titers of IgG2a antibodies, particularly in the i.p. alum-based model, might indicate a mixed Th1/Th2 response of the immune system.

Next, we characterized cytokine profiles in sera of mice sensitized to the soybean Gly m 4 allergen via i.p. or s.c. routes in order to provide further insights into the immunological mechanisms underlying sensitization to Gly m 4. In adjuvant-free models we observed a significant reduction in pro-inflammatory cytokines, such as IL-1α and IL-6. Recently, it was shown that IL-6 prevents cell polarization towards Th2 by promoting SOCS3-dependent suppression of IL-2 signaling [19]. Also, we observed a mild increase in Th2-associated IL-13 and GM-CSF, particularly in the i.p. group. Previously, it has been demonstrated that IL-13, one of the key cytokines responsible for allergic sensitization, shares many biologic activities with IL-4 and is required for induction of allergen-specific IgE [20]. The decrease in the production of chemokines, such as CCL11, CCL5, and CXCL1, in adjuvant-free models may indicate impaired leukocyte recruitment, which can potentially lead to reduced inflammatory cell infiltration during sensitization. The obtained results suggest that the soybean allergen Gly m 4 alone in the absence of adjuvant can modulate immune response by suppressing acute inflammatory responses and promoting a Th2-skewed environment.

When compared to adjuvant-free models, Th2-mediated responses were more pronounced in mice sensitized to Gly m 4 with aluminum hydroxide. For instance, not only IL-13, but also IL-5, a cytokine playing a crucial role in allergic reactions by promoting the development, maturation, and activation of eosinophils, was significantly elevated in sensitized mice, particularly via the i.p. route [21]. Eotaxin-1/CCL11, which plays a significant role in attracting eosinophils and Th2 cells to sites of allergic inflammation, was also increased in the sera of intraperitoneally sensitized mice [22]. The obtained results indicate that, while aluminum hydroxide acts as an adjuvant by amplifying the immune response and promoting stronger Th2-mediated inflammation, the soybean allergen Gly m 4 alone is sufficient to induce sensitization, at least via i.p. or s.c. administration routes.

Thus, in our study, all four models (i.p. and s.c. with or without alum) led to successful sensitization of mouse immune system to Gly m 4, with life-threatening systemic allergic reactions upon challenge. While alum-based models were characterized by higher Gly m 4-specific IgE and IgG1 titers and their levels were more homogeneous (Figure 1c,d), adjuvant-free models seem to be more physiological in terms of induction of sensitization. Based on the cytokine levels in sera of sensitized mice, i.p. administration seems to induce more systemic immune response compared to s.c. one. Our data are in line with a previous study, where i.p. route of the birch Bet v 1 allergen exposure during sensitization induced more systemic response, while s.c. route predominantly led to local Th2 response [16]. A limitation of our study that has to be mentioned is that i.p. and s.c. routes do not fully mimic the physiological way of food allergen exposure, such as oral ingestion, which involves digestion and interaction with the intestinal epithelium [23]. Moreover, we have demonstrated that birch pollen Bet v 1, the major human sensitizer, elicited a more moderate immune reaction than soybean Gly m 4, which may highlight that the immunogenicity of an allergen in a mouse model does not always directly correlate with its clinical significance in humans. And this limitation is common to many allergy models. Future studies should explore oral sensitization models to better display human exposure to the soybean allergen Gly m 4.

## 4. Materials and Methods

### 4.1. Production of Recombinant Allergens

His-tagged recombinant allergens Gly m 4 and Bet v 1 were produced by heterologous expression in the ClearColi^®^ BL21(DE3) strain of *Escherichia coli* and purified as previously described [8]. The purified proteins were characterized by SDS-PAGE, MALDI-TOF mass spectrometry, and circular dichroism spectroscopy as previously described [8]. For the sensitization step, intact Gly m 4 and Bet v 1 allergens after removal of octahistidine tag were used. For the challenge in already sensitized to Gly m 4 and Bet v 1 mice, we used His-tagged allergens. The final stage of purification in the case of all proteins was RP-HPLC. The purity of the obtained allergens according to RP-HPLC and SDS-PAGE was assessed as >98%.

### 4.2. Mice

Specific pathogen-free (SPF) female BALB/c mice (6–8 weeks old) were obtained from the Department of Experimental Biology with Vivarium, Shemyakin–Ovchinnikov Institute of Bioorganic Chemistry, RAS (Moscow, Russia). Mice were housed under pathogen-free conditions and maintained with a 12 h light/dark cycle. Prior to the study, animals were acclimated for one week. All animal experiments were approved by the local Ethics Committee.

### 4.3. Sensitization Protocols and Allergic Challenge

Mice were randomly divided into 10 groups (n = 5 per group). Sensitization protocols included 4 subcutaneous or intraperitoneal injections of 5 μg of recombinant Gly m 4, either adjuvant-free or adsorbed onto 1 mg of aluminum hydroxide adjuvant (Alhydrogel^®^, 2%; InvivoGen, San Diego, CA, USA), administered in 150 μL of PBS (pH 7.4) per mouse on days 0, 14, 28, and 42 (Figure 1a). For comparison, additional groups of mice were sensitized via subcutaneous or intraperitoneal injections according to the same schedule with 5 μg of recombinant birch Bet v 1 adsorbed onto 1 mg of alum. Control groups received 150 μL of PBS or 1 mg of alum in PBS alone on the same days. Two separate groups were sensitized intranasally with six administrations (12 μL each) of 20 μg of purified Gly m 4, either alone or in combination with 20 ng of lipopolysaccharide (LPS) from *Escherichia coli* O111:B4 (cat. #L2630, Merck, Darmstadt, Germany) as an adjuvant, on days 7, 14, 21, 28, 35, and 42 (Figure 1b). On days 49 and 51, all mice were challenged via intraperitoneal injection with 100 μg of Gly m 4 or Bet v 1 in 150 μL of PBS per mouse. Control mice were challenged with PBS. Systemic anaphylaxis reactions were evaluated 20–40 min after challenge according to a scoring system [24]: 0, no symptoms; 1, scratching of ear and mouth; 2, puffiness around eyes and mouth, pilar erection, labored breathing; 3, cyanosis around the mouth and tail; 4, severely reduced motility, tremors, severe respiratory distress; 5, death. Blood samples were collected on days −3 (preimmune), 48 (one week after the last sensitization), and 58 (one week after the final challenge), and analyzed for allergen-specific IgE, IgG1, and IgG2a antibodies.

### 4.4. Determination of Allergen-Specific IgE, IgG1, and IgG2a by ELISA

Levels of Gly m 4- and Bet v 1-specific IgE, IgG1, and IgG2a antibodies in mouse sera were evaluated by enzyme-linked immunosorbent assay (ELISA). High-binding flat-bottom 96-well microplates (Jet Biofil, Guangzhou, China) were coated with recombinant allergens (0.5 μg/well) in phosphate-buffered saline (PBS, pH 7.4) and incubated for 1 h at 37 °C. Then the plates were blocked with 2% bovine serum albumin (BSA; SERVA, Heidelberg, Germany) in PBS for 1 h at 37 °C in order to prevent nonspecific binding. Following blocking, wells were incubated overnight at 4 °C with preimmune or immune mouse sera, diluted in PBS with 0.5% BSA. Dilution factors were 1:10 for IgE detection, 1:50 for IgG1, and 1:20 for IgG2a. The following day, plates were washed and incubated for 2 h at 37 °C with biotinylated monoclonal anti-mouse antibodies specific for IgE (1:2000), IgG1 (1:1000), or IgG2a (1:10,000) (all from Invitrogen, Waltham, MA, USA), diluted in PBS containing 0.5% BSA. After washing, the wells were incubated at 37 °C with streptavidin conjugated to horseradish peroxidase (HRP; 1:10,000 dilution, Thermo Scientific, Rockford, IL, USA). After washing, TMB substrate (Macklin, Shanghai, China) was added to initiate the enzymatic reaction. Then the reaction was stopped with 2 M H_2_SO_4_ and the absorbance was measured at 450 nm. Each experimental condition was assessed in quadruplicate (four technical replicates), and all experiments were independently repeated at least twice to ensure reproducibility.

### 4.5. Degranulation of RBL-2H3 Cells

The rat basophilic leukemia cell line RBL-2H3 (ATCC CRL-2256) was cultured in complete DMEM/F12 (1:1) medium (Gibco, Waltham, MA, USA) supplemented with 10% fetal bovine serum (FBS; Capricorn Scientific, Ebsdorfergrund, Germany) and 1× antibiotic-antimycotic solution (Invitrogen, Waltham, MA, USA) in a humidified CO_2_-incubator (5% CO_2_, 37 °C). RBL-2H3 cells (5 × 10^5^/mL) were seeded into the wells of 96-well plate (200 μL/well) and incubated overnight. On the following day, cells were passively sensitized with pooled serum of mice within the same experimental group. For the sensitization, the medium was replaced with DMEM/F12 containing 1% pooled mouse serum and 1× antibiotic-antimycotic solution, followed by incubation for 16–18 h in a humidified CO_2_-incubator. Pooled serum from PBS-sensitized mice served as a negative control. After the sensitization, the cells were washed three times with Tyrode buffer A (135 mM NaCl, 5 mM KCl, 1.8 mM CaCl_2_, 1 mM MgCl_2_, 10 mM HEPES, 5.6 mM D-(+)-glucose, 0.1% [*w*/*v*] BSA; pH 7.4). Cells were then stimulated with serial 10-fold dilutions of Gly m 4 (0.01–10 μg/mL) in Tyrode buffer A for 1.5 h at 37 °C. Wells without antigen served as controls for monitoring spontaneous β-hexosaminidase release. Total enzyme release was determined by adding 0.1% Triton X-100 to the cells. After stimulation, 50 μL of supernatant was transferred to a new plate and mixed with 30 μL of substrate solution (4 mM 4-nitrophenyl-N-acetyl-β-D-glucosaminide (MedChemExpress, South Brunswick, NJ, USA) in citrate buffer, pH 4.5), followed by incubation for 1.5 h at 37 °C. The enzymatic reaction was stopped by adding 120 μL of stopping buffer (0.1 M glycine-NaOH, pH 10.0). Absorbance was measured at 405 nm using a UV/Vis PlateReader AF2200 (Eppendorf, Hamburg, Germany). Degranulation was quantified as the percentage of β-hexosaminidase released relative to total enzyme content.

### 4.6. Assessment of Cytokine Profiles in Mouse Sera by Multiplex Technology

The levels of 48 analytes were quantified using the MILLIPLEX Mouse Cytokine/Chemokine/Growth Factor Panel kit (cat. #MCYTMAG-70K-PX32, Merck, Darmstadt, Germany) with multiplex xMAP technology (Luminex, Austin, TX, USA). The analytes included Eotaxin/CCL11, G-CSF, GM-CSF, IFN-γ, IL-1α, IL-1β, IL-2, IL-3, IL-4, IL-5, IL-6, IL-7, IL-9, IL-10, IL-12 (p40), IL-12 (p70), IL-13, IL-15, IL-17, IP-10/CXCL10, KC/CXCL1, LIF, LIX/CXCL5, MCP-1/CCL2, M-CSF, MIG/CXCL9, MIP-1α/CCL3, MIP-1β/CCL4, MIP-2/CXCL2, RANTES/CCL5, TNF-α, and VEGF. The assay was conducted on the MAGPIX platform (Merck) using xPONENT 4.2 software (Merck), following the manufacturer’s protocol, which involved overnight incubation of samples with primary antibodies. Final data analysis was performed using MILLIPLEX Analyst v5.1 software (Merck). Each sample was measured in duplicate.

### 4.7. Statistical Analysis

Immunoglobulin levels in mouse sera before and after sensitization were compared using the Wilcoxon matched-pairs signed-rank test. β-Hexosaminidase release in passively sensitized RBL-2H3 cells in response to 10-fold serial dilutions of Gly m 4 was analyzed using multiple *t*-tests with False Discovery Rate (FDR) correction according to the Benjamini-Krieger-Yekutieli procedure. Comparisons of cytokine levels in sera and clinical score of systemic anaphylactic reactions between control and experimental groups of mice were performed using Mann–Whitney U test and Kruskal–Wallis test with Benjamini–Krieger–Yekutieli FDR correction. All statistical analyses were conducted using GraphPad Prism v.8.0.1 (GraphPad Software, Inc., San Diego, CA, USA). The *p*-values ≤ 0.05 were considered significant.

## 5. Conclusions

Our study reported the development of adjuvant-free murine models of sensitization to the soybean allergen Gly m 4, associated with severe manifestations of a soy allergy, including life-threatening systemic reactions. The lack of necessity to use an adjuvant for the development of Th2-type responses demonstrated intrinsic sensitization potential of Gly m 4 in vivo when administered via intraperitoneal or subcutaneous injections. These findings suggest that in patients with a birch-related allergy to soy, the allergic reactions may be driven not only due to cross-reactivity with Bet v 1, but also due to concomitant true sensitization to Gly m 4. This should be kept in mind when prescribing birch pollen extracts or hypoallergenic analogs of Bet v 1 during allergen-specific immunotherapy (AIT) in such patients, since immunotherapy may be ineffective in reducing allergic reactions to soy [25]. The established models in our study provide a valuable platform for testing novel approaches of AIT to treat a birch-related allergy to soy.

## Data Availability

All data generated and analyzed during this study are included in this published article and its Supplementary Materials.

## Supplementary Materials

The following supporting information can be downloaded at: https://www.mdpi.com/article/10.3390/ijms262311695/s1.

## Abbreviations

The following abbreviations are used in this manuscript:

AIT	Allergen-specific immunotherapy
ELISA	Enzyme-linked immunosorbent assay
LPS	Lipopolysaccharides from *E. coli*
PBS	Phosphate-buffered saline
RBL	Rat basophilic leukemia
