# Supplementary material for: Adjuvant-Free Murine Models of Allergic Sensitization to the Major Soybean Allergen Gly m 4
Source: Int J Mol Sci. 2025 Dec 3;26(23):11695. doi: 10.3390/ijms262311695 (PMC12692166; doi:10.3390/ijms262311695)
Supplement: Supplementary file 1 [file ijms-26-11695-s001.zip › Supplementary Figure S1.docx]

**
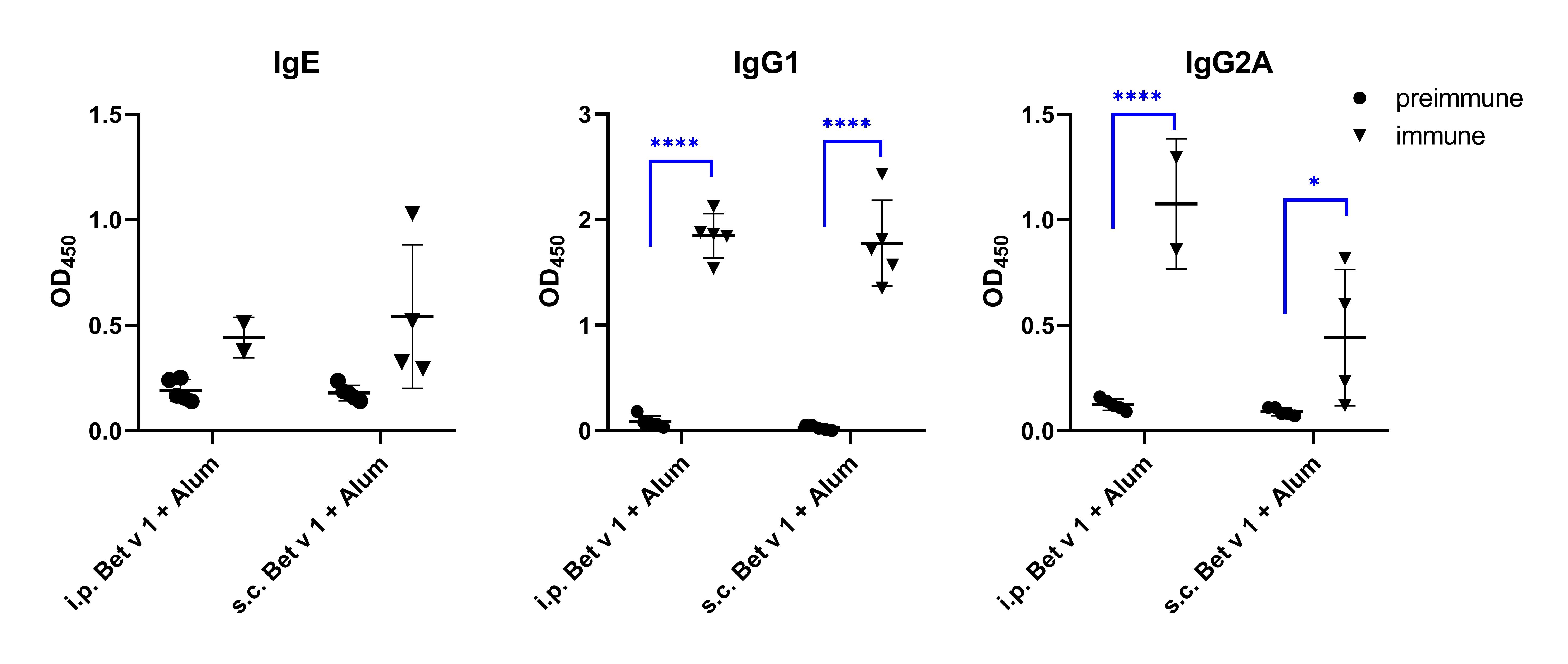
**

**Figure S1:** Levels of Bet v 1-specific IgE, IgG1, and IgG2a in mice sera before (preimmune) and after (immune) sensitization via intraperitoneal (i.p.) or subcutaneous (s.c.) Bet v 1 administration with adjuvant alum according to the same protocol as for Gly m 4.
